# Developmental Considerations in How Defense Attorneys Employ Child Sexual Abuse and Rape Myths When Questioning Alleged Victims of Child Sexual Abuse

**DOI:** 10.1177/08862605231189512

**Published:** 2023-08-02

**Authors:** Emily Denne, Suzanne St. George, Stacia N. Stolzenberg

**Affiliations:** 1 Griffith University, Brisbane, QLD, Australia; 2 University of Arkansas at Little Rock, USA; 3 Arizona State University, Phoenix, USA

**Keywords:** child abuse, sexual abuse, rape myths, child sexual abuse myths

## Abstract

Myths and misconceptions surrounding the nature of sexual assault play a role in shaping the perceptions of victims as credible and perpetrators as culpable. Defense attorneys often capitalize on myths in court as an element of their defense strategies. Researchers have established that myths about both rape generally, and child sexual abuse (CSA) specifically, appear with regularity in criminal trials of children who have made an allegation of CSA. Yet no work has systematically and quantitatively examined the impact of a child’s age on the probability that attorneys will ask a myth-consistent question in criminal trials of CSA. In the current study, we examine 6,384 lines of questioning across 134 criminal trials of CSA to assess whether defense attorneys employ developmentally sensitive strategies when asking children questions that draw upon myths about sexual violence (CSA myths: disclosure myths, extent of harm, a child’s positive relationship with their perpetrator, and the presence of witnesses; Rape myths: force and resistance, motives to lie, victim precipitation, and character issues). We found that attorneys did not vary their use of *CSA myths* by the age of the child. However, the probability that a child would receive a *rape myth*-consistent line of questioning, increased with a child’s age. This work suggests that attorneys are, at times, strategic in their use of myths and employ these adult rape myths in ways that are plausible, purposeful, and likely impactful. The strategic use of these questions may acknowledge young children’s limited development but may place too great a demand on older children’s developmental capacities. Prosecutors should be prepared to counterquestion these myths in redirect examination.

Myths and misconceptions about sexual violence have widespread repercussions for sexual assault case processing. Scholars have consistently documented that *rape myths* shift focus from perpetrators’ responsibility and aggression to victims’ responsibility and credibility (Dinos et al., 2015; Kopper, 1996; Lonsway & Fitzgerald, 1994; Suarez & Gadalla, 2010). Unsurprisingly, therefore, researchers find that rape myths are impactful and shape perceptions of victim credibility and perpetrator accountability at every stage of the investigation and prosecution process (Hayes et al., 2013; Hine & Murphy, 2019; Rich & Seffrin, 2012).

Much of the existent research on rape myths explores the role of rape myths in sexual assault cases involving adults (Smith & Skinner, 2017), with a smaller body of work exploring the use of rape myths in cases of child sexual abuse (CSA). Importantly, researchers define and differentiate CSA myths, which apply specifically and more exclusively to sex crimes involving children, compared to adult rape myths (Cromer & Goldsmith, 2010). Both rape myths and CSA myths appear with regularity in the cross-examination of child witnesses in CSA cases (Denne et al, 2023; St. George, Denne, et al., 2022). Yet, no work to date has explored if an attorney’s use of rape myths and CSA myths in cross-examination varies strategically and systematically as a function of children’s age. As children, particularly very young children, do not have the linguistic abilities nor the developmental maturity to recognize and combat myths in court, it is important to identify attorneys’ strategies when employing these myths to better arm prosecutors to dismantle them.

Our research draws upon literature on developmentally sensitive questioning in CSA cases, combined with literature on the use of myths in attorney questioning. We were interested in developmental considerations in the use of rape and CSA myths in defense attorney questions to CSA victims. Since researchers find that attorneys, at times, employ some age-sensitive strategies when questioning CSA victims in court (Stolzenberg & Lyon, 2014), we would expect attorneys to be somewhat discerning in how they use rape myths and CSA myths when questioning child victims. Specifically, we use a sample of 134 transcripts of trial testimony from CSA cases tried in a large Southwestern jurisdiction in the United States to examine how attorneys vary their use of myths about sexual violence by the age of the alleged victim. Both rape and CSA myths shape perceptions of victim credibility (Hayes et al., 2013), which subsequently shape case processing and case outcomes (Dinos et al., 2015; Morabito et al., 2019) with tangible implications for child safety and public health broadly. As such, this work is a critical step in documenting the content of defense attorney questions and the ways in which children’s developmental capacities inform their strategies in court.

## Age-Sensitive Questioning Strategies

The accuracy and consistency of a child’s statements in court are shaped by their own memory, developmental capacity, and language ability (Klemfuss & Ceci, 2012). The accuracy and consistency of their statements also are shaped by the content and structure of attorney questions which serve to exploit or accommodate children’s vulnerabilities as young witnesses (Brown & Lamb, 2015). Researchers in the field of investigative interviewing have documented the ways in which children’s memory and linguistic skills situate them as vulnerable witnesses (Bottoms et al., 2009; Lamb et al., 1994; Pipe et al., 2004). First, early studies on children’s memory suggest that as a child’s age increases so does their ability to remember (for a review see Schneider & Ornstein, 2015). While young children can remember less, their memories tend to be generally accurate (Goodman & Reed, 1986). Still, the ways in which questions are posed to young children (e.g., suggestive or linguistically complex questions) impact their recall accuracy more strongly than adults (Cassel et al., 1996). Second, children’s linguistic skills limit their ability to communicate their experiences when compared to adults. As a child’s age increases so does the length and detailedness of their narrative reports (Nelson, 1993). Furthermore, young children often lack the vocabulary to provide descriptions (e.g., the use of adjectives/adverbs; descriptions of emotion) to describe past events in the same level of detail as adults (Barrett, 1995; Peterson & Biggs, 2001). In this way, both children’s limited memory for events and ability to communicate those memories make the ways in which we talk to them about their experiences important for eliciting detailed, reliable, and accurate reports. It is not surprising that attorneys will be sensitive to children’s developmental trajectories and use questioning to minimize (as prosecutors) or capitalize on (as defense attorneys) children’s vulnerabilities. Attorneys may tailor their use of myths based on their knowledge of child development and their motivations to minimize or capitalize on child vulnerabilities.

## Attorney Questioning Strategies

Evidence suggests that defense and prosecuting attorneys talk to children differently. Attorneys are sensitive to the developmental variation of child witnesses, and they sometimes vary the structure and content of their questions in ways that reflect developmental trajectories (Denne et al., 2020; St. George et al., 2022; Stolzenberg et al., 2020). For example, Stolzenberg et al., (2020) found that defense attorneys asked more yes/no questions to younger children, whereas prosecutors did not vary the use of yes/no questions by the child’s age. By contrast, Klemfuss et al. (2017) found that prosecutors incorporated more temporal structure in questioning than defense attorneys, and that a child’s age influenced the temporal structure of prosecutors’ questions, but not defense attorneys’ questions. In addition, researchers find that a greater proportion of prosecutors’ questions, compared to defense attorneys’ questions, are open-ended or wh-/how questions, which are less suggestive and easier for children to understand (Stolzenberg & Lyon, 2014). Furthermore, when asking about complex or confusing topics, prosecutors do so more directly. In a study assessing attorneys’ questioning about suggestive influences, for example, St. George, Sullivan et al. (2022) found that prosecutors asked more direct questions about suggestive influence than defense attorneys, while defense attorneys asked more subtle and suggestive questions, including questions with multiple meanings that children struggle to understand. Similarly, Sullivan et al., (2022) found that defense attorneys’ use of suggestive questioning styles resulted in more instances of children misunderstanding than the questioning styles of prosecutors. Collectively, therefore, researchers find that prosecutors are more sensitive to children’s developmental limitations and incorporate more evidence-based practices (such as using more open-ended questions) for questioning children in court than defense attorneys.

## The Impact of a Child’s Age on Perceptions of Child’s Credibility

Researchers find that the victim’s age influences perceptions of and responses to sexual violence. In studies assessing perceptions of hypothetical CSA incidents, scholars frequently find that observers perceive victims as more credible and perpetrators as more culpable when the victims are younger, though this relationship may not be linear. For example, scholars found that mock jurors perceived 5-year-old victims (Davies & Rogers, 2009) and 10-year-old victims (Rogers & Davies, 2007; Rogers et al., 2007) as more credible than 15-year-old victims in depicted stranger and familial CSA scenarios. By contrast, Castelli et al. (2005) found no difference in perceived perpetrator guilt across scenarios depicting 4- and 7-year-old victims. Furthermore, researchers found that extralegal factors like child attractiveness, prior CSA victimization, a lack of resistance, and the child–perpetrator relationship influence perceived victim blame and credibility in depicted scenarios involving children as young as 10 years old (Rogers & Davies, 2007; Rogers et al., 2007). As such, rape myths may interact with age in jurors’ assessments of victim credibility and perpetrator guilt. Even though consent is not a legitimate defense in CSA cases, jurors may perceive older children and teenagers as more autonomous or consenting to sex acts, particularly if abuse involves grooming and seduction, no force or resistance, multiple incidents, and a positive child–perpetrator relationship. There may be some age threshold at which point myths framing sexual violence as consensual come into play, and attorneys may vary their questioning strategies in ways that account for the child’s age.

## Rape and CSA Myths

The effects of rape myths and CSA myths on perceptions and responses to sexual violence are well documented. Researchers consistently find that rape myths and CSA myths influence perceptions of hypothetical cases, including assessments of victim responsibility and credibility (Bottoms et al., 2017; Grubb & Turner, 2012; van der Bruggen & Grubb, 2014; Suarez & Gadalla, 2010). For example, scholars frequently find that observers blame victims more and perpetrators less, when the victim is perceived to precipitate the assault, such as by being intoxicated (Grubb & Turner, 2012). In this way, victims who consume drugs or alcohol are viewed as taking risks and precipitating abuse. Similarly, responding to a hypothetical CSA incident, observers perceived victims as more credible when they were assaulted by a stranger, compared to their father or a family friend (Davies & Rogers, 2009). Rape myths are also evident in criminal justice responses to real cases. For example, cases that do not involve force are significantly more likely to result in an acquittal than cases that do (Stolzenberg & Lyon, 2014). Furthermore, scholars found that myths and misconceptions come up in jury deliberations (Ellison & Munro, 2010) and influence jury verdicts (Dinos et al., 2015). For instance, when given the opportunity to ask questions to children testifying about CSA, jurors commonly question children about rape and CSA myths, such as lack of resistance or delayed disclosures (St. George et al., 2020).

Building upon this work involving rape myths, Denne et al. (2023) hypothesized that CSA myths would appear in the questioning of CSA victims in court. The authors coded for myths related to the disclosure of abuse, witnesses and privacy, assumptions of harm, and the child’s positive relationship with their perpetrator. These myths appeared in over 10% of defense attorney lines of questioning asked to children in their sample. While it may be plausible for attorneys to draw upon CSA myths in CSA cases, as they tend to reflect misunderstandings about the nature of child abuse and abuse disclosure, evidence suggests that attorneys draw upon adult rape myths in CSA cases as well. St. George, Denne, et al. (2022) explored the presence of rape myths related to force and resistance, motives to lie, victim precipitation, and character issues in defense attorney questioning of CSA victims. Again, about 10% of all defense attorney lines of questioning drew upon a rape myth. Combined, these findings suggest that both rape and CSA myths appear with some degree of regularity in the questioning of children alleging sexual violence. Yet little work has documented the ways in which attorneys vary the use of both CSA and rape myths strategically as a function of the child’s age.

## Attorney Questioning, Child’s Age, and Myths

Attorneys’ questioning strategies are likely shaped by their motivations and roles in court and informed by their knowledge of child development. Evidence suggests that both prosecuting and defense attorneys are educated and informed about the best questioning strategies to use with young children (i.e., using open-ended questions; Bruer et al., 2022). Yet, prosecuting attorneys report *utilizing* best questioning practices with children more often than defense attorneys (Bruer et al., 2022). This suggests that attorneys’ motivation guides their questioning practices more than their knowledge of best practice. We would expect the same to be true when examining question content in addition to question style. That is, defense attorneys are likely aware of rape and CSA myths and their use of these myths may be guided by their motivations in court. A child’s age may then inform the use of these myths as attorneys will be interested in drawing upon myths that reflect probable age-related trends or age-related (mis)perceptions of jurors.

## The Current Study

Given evidence that defense attorneys tailor the content and style of their questioning to capitalize on children’s developmental vulnerabilities, coupled with evidence that myths impact perceptions of child credibility, we expected defense attorneys’ strategic use of rape and CSA myth questioning would likewise vary with child age. As CSA and rape myths are distinct, myths vary by age, so we expect that the *use* of myths will also vary by age. We hypothesized that:

*H1*: Attorneys would ask more CSA myth questions to younger children because scholars frequently find that observers perceive victims as more credible and perpetrators as more culpable, when the victims are younger (Davies & Rogers, 2009; Gabora et al., 1993; Rogers & Davies, 2007). Defense attorneys may try to combat this tendency by highlighting CSA-specific myths and misconceptions to discredit child victims, particularly young victims. We expected that the probability that a child would receive a CSA myth question would decrease with age.
*H2*: We expected that defense attorneys would ask more rape myth questions to older children since there is some evidence that jurors perceive older children as more autonomous. We expected that the probability a child would receive a rape myth question would increase with age.

In addition to these hypotheses, we conducted a few exploratory analyses. We explored if there was a particular age at which the attorneys’ shifted their questioning strategy from CSA myths to rape myths by assessing if there was a nonlinear relationship between age and question type. Finally, we conducted an exploratory analysis to assess differences in attorneys’ use of *specific* CSA and *specific* rape myths by the age of the child. Across all analyses, we controlled for the impact of abuse severity, abuse frequency, a child’s relationship to their perpetrator, and child gender, as all are related to how children disclose and describe abuse (Hershkowitz et al., 2007; Malloy et al., 2007).

### Methods and Procedure

Our sample consisted of transcripts of 134 testimonies from criminal trials of CSA occurring between January 2005 and December 2015 in Phoenix, Arizona. Data were collected in the United States. We contacted and paid court reporters to provide us with transcripts of complete cases. We had a 64% response rate. Cases were included if they had complete transcripts, involved a minor, and involved at least a single charge of Sexual Conduct with a Minor (A.R.S. 13-1405), Child Molestation (A.R.S. 13-1410), or Sex Abuse (A.R.S. 13-1404). The children included in this sample ranged in age from 5 to 17 years (*M* = 12.48, *SD* = 3.34). The majority of defendants were male (99%) and the majority of victims were female (90%). In many of the cases the defendant was the child’s parent or caregiver (40%). In the remaining cases the defendant was either another family member (26%), a family friend or familiar adult (29%), or a stranger (5%). Children’s allegations in these cases included penetration (34%), oral copulation or genital contact (14%), and less severe abuse such as exposure to pornography (52%). We excluded the testimonies of four children who were questioned by the defendant and eight children for whom the defense declined to ask any questions, resulting in a total of 122 testimonies across 77 defense attorneys.

### Thematic Analysis and Reliability

Two independent coders coded each transcript of the defense cross-examination of the witness. Authors coded for lines of questioning which were defined as a series of question–answer pairs that developed a particular theme or topic (such as a line of questioning centering around a prior disclosure; see St. George, Denne, et al., 2022 for detailed description of lines of questioning coding). Lines of questioning did not always refer specifically to different types of myths, but instead focused on a topic or theme within a line of questions. Across the 122 transcripts there were a total of 6,384 lines of questioning (range: 1–185, *M* = 52 per child; *SD* = 37.3 per child). Two independent coders read through each transcript to identify lines of questioning. They then met to resolve any discrepancies and reached 100% agreement across the sample.

Coders then used thematic analysis to develop a coding guide covering issues related to CSA myths (disclosure, witnesses and privacy, assumptions of harm, and the child’s relationship with their perpetrator) as well as rape myths more generally (force and resistance, motives to lie, precipitation, and character issues; see Table 1 for coding definitions).

**Table 1.** CSA Myth and Rape Myth Definitions.

Category	*N* (%) of all lines of questioning	Definition
**CSA myths**	**674 (10.6)**	
Disclosure	257 (4.0)	Highlight a failure to disclose, delay in disclosure, partial disclosure, and denials of abuse.
Witnesses and privacy	207 (3.2)	Emphasized or suggested that others were nearby or present during abuse episodes and so could have noticed/seen/witnessed abuse if something was going on. Included questions about the perpetrators’ attempts to make the abuse private.
Assumptions of harm	142 (2.2)	Emphasized either physical or emotional harm or lack of harm resulting from the abuse or disclosure of the abuse.
Child’s positive relationship	77 (1.2)	Positive-valence terms to describe the child and perpetrator’s relationship.
**Rape myths**	**681 (10.7)**	
Force and resistance	189 (3.0)	Child verbal or physically resistance to abuse, defendants force or a lack of force, and the defendant asking or threatening the child not to disclose.
Motives to lie	178 (2.8)	Reasons a child would lie about abuse to cover up a transgression, get revenge, or other motives.
Precipitation	173 (2.7)	Risky behavior that precipitated the abuse such as provocative clothing, drug use, promiscuity, and so on.
Character issues	155 (2.4)	Character issues associated with the child’s credibility such as behavioral problems (e.g., habitual lying) and criminal behavior.

All categories were coded and recoded, calculating Kappa periodically. Coding continued until coders reached reliability (*K* > 0.80 on all variables). All discrepancies were then resolved so there was perfect agreement across all variables. Qualitatively, these myths are described in great detail in St George, Denne, et al. (2022) and Denne et al. (2023). The present article serves to expand upon these published pieces by quantitatively exploring age differences in how defense attorneys use these myths.

## Results

We first tested Hypothesis 1, where we expected the probability that a child would receive a CSA myth question to decrease with age. We examined the probability that a child would receive a *CSA myth* line of questioning by the child’s age using generalized linear mixed-effects models (GLMMs). Age (continuous) was used as our independent variable and the presence of a CSA myth in each line of questioning asked to the child was our dependent variable (0 or 1). Our GLMM, using a binary response variable and a logistic link function, included a by-subject random intercept as each child received multiple lines of questioning. We controlled for the child’s gender, the frequency of abuse, severity of abuse, and their relationship to their perpetrator with fixed effects. The overall model was significant (*F*[10, 5055] = 2.23, *p* = .014) with abuse severity (*F*[1, 5055] = 3.95, *p* = .019) predicting the presence of a CSA myth. There was no significant relationship between the child’s age and the presence of a CSA myth, *F*(1, 5055) < 0.01, *p* = .821, indicating that Hypothesis 1 was not supported. We then tested for a nonlinear relationship between age (using an age-squared term) and the probability that a child would receive a CSA myth line of questioning using GLMMs to explore whether there was a particular age at which point the attorneys’ shifted their questioning strategy from CSA myths to rape myths. There was no significant nonlinear relationship between the child’s age and the probability that a child would receive a CSA myth line of questioning, *F*(1, 5055) = 0.849, *p* = .357.

Next, we tested Hypothesis 2 where we expected that the use of rape myths would increase with the child’s age. We replicated the analyses used to assess CSA myth questioning, to analyze rape myth questioning. Again, we used GLMMs with rape myth questioning as a binary dependent variable and age as a continuous independent variable, and we controlled for the child’s gender, abuse frequency and severity, and the child–defendant relationship. The overall model was significant (*F*[20, 5055)] = 2.131, *p* = .002) with age (*F*[1, 5055] = 9.67, *p* = .003) serving as a significant predictor of the probability that a child would receive a rape myth line of questioning. This indicated support for Hypothesis 2. As the child’s age increased, so did the probability of a rape myth line of questioning (*B* = −0.14, *SE* = 0.05, *p* = .003, odds = 0.87, 95% CI [−0.235, −0.015]; see Table 2 for descriptives). However, the nonlinear relationship between age and rape myth questioning was not significant, *F*(2, 5055) = 0.859, *p* = .454.

**Table 2.** Descriptive Statistics Exploring the Relationship Between the Child’s Age and the Proportion of Rape Myth Lines of Questioning.

Age Range	Range	*M*	*SD*
5–10	0.0–0.15	0.039	0.045
11–14	0.0–0.29	0.073	0.66
15–17	0.0–0.50	0.110	0.89

*Note*. Age binned for the purpose of descriptive analysis (see Denne et al., 2019), generalized linear mixed-effects model (GLMM) was conducted with age entered as a continuous variable.

To explore difference in attorney’s use of *specific* CSA and *specific* rape myths by the age of the child, we then conducted a series of GLMMs exploring both a linear and nonlinear relationship between the child’s age and each *specific* CSA myth (disclosure, witnesses and privacy, assumptions of harm, and child’s positive relationship with their perpetrator) and each *specific* rape myth (force and resistance, motives to lie, precipitation, and character issues). Again, our GLMMs used a binary response variable and a logistic link function and included a by-subject random intercept as each child received multiple lines of questioning. We controlled for the child’s gender, the frequency of abuse, severity of abuse, and their relationship to their perpetrator. Results from these models are presented in Table 3.

**Table 3.** GLMMs Exploring a Linear and Nonlinear Relationship Between the Child’s Age and Each Subcategory of CSA and Rape Myths.

Myth Category	GLMM Linear Relationship					GLMM Nonlinear Relationship				
	β	*SE*	Odds	*p*	95% CI	β	*SE*	Odds	*p*	95% CI
All myths	−.068	0.024	0.93	.005*	[−.086, 6.74]	−.006	0.008	1.00	.422	[−0.021, 0.009]
CSA myth	−.007	0.030	0.99	.812	[−0.066, 0.051]	.009	0.009	1.00	.357	[−0.010, 0.028]
Disclosure	−.071	0.068	0.93	.295	[−0.205, 0.062]	.025	0.024	1.03	.290	[−0.021, 0.071]
Witnesses and privacy	.074	0.048	1.08	.127	[−0.021, 0.168]	.021	0.018	1.02	.250	[−0.015, 0.057]
Assumptions of harm	−.050	0.063	0.95	.427	[−0.173, 0.073]	−.018	0.016	0.98	.261	[−0.049, 0.013]
Child’s positive relationship	0.049	0.064	1.05	.438	[−0.076, 0.174]	.007	0.023	1.01	.751	[−0.038, 0.053]
Rape myths	−.143	0.046	0.87	.003*	[−0.235, −.051]	−.143	0.046	0.867	.442	[−0.235, −0.051]
Force and resistance	−.137	0.073	0.06	.872	[−0.281, 0.006]	.015	0.023	1.02	.551	[−0.031, 0.061]
Motives to lie	−.071	0.077	0.93	.360	[−0.222, 0.081]	−.025	0.018	0.975	.151	[−0.060, 0.009]
Precipitation	−.141	0.092	0.87	.126	[−0.322, 0.040]	−.028	0.023	0.972	.214	[−0.072, 0.016]
Character issues	−.232	0.068	0.79	< .001*	[−0.364, −0.099]	−.021	0.015	0.980	.983	[−0.049, 0.008]

*Note*. GLMM = generalized linear mixed-effects model.

Overall, more specific myth questioning was not related to age, either linearly or nonlinearly. With the exception of character issus, age was not related to any of the specific CSA myth questions or rape myth questions explored.

## Discussion

In the present study, we systematically explored whether defense attorneys employed both rape myths and CSA myths in age-sensitive ways when questioning children in CSA criminal trials. In doing so, we found support for some, but not all, of our hypotheses. Specifically, we found that attorneys employed rape myths, but not CSA myths, in ways that were predictable, strategic, and plausible. That is, the probability that a child would receive a rape myth-consistent line of questioning increased as the age of the child increased. Our work expands upon prior work examining myths about sexual violence as well as work examining developmentally sensitive questioning in court.

### Effects of the Child’s Age by CSA and Rape Myths

Across CSA myths (which drew upon myths related to disclosure, witnesses, and privacy, assumptions of harm, and a child’s positive relationship with their perpetrator), defense attorneys did not vary their questioning content in ways that were strategic or developmentally sensitive. We expected that defense attorneys would be more likely to ask CSA myths to younger victims compared to older victims. This was because researchers often find that observers perceive victims as more credible and perpetrators as more culpable, when the victims are younger (Davies & Rogers, 2009; Gabora et al., 1993; Rogers & Davies, 2007). We expected attorneys to try to combat this tendency by highlighting CSA-specific myths and misconceptions to discredit young victims. Instead, the age of the child did not predict attorneys’ use of CSA myths and children in every age group received CSA-myth consistent questions. This finding likely reflects a lack of sensitivity to developmental considerations and young children’s rudimentary ability to identify and combat questions that draw upon myths about sexual violence and highlight an ambiguity of blame. Furthermore, defense attorneys may be willing to use any strategy, such as drawing upon CSA myths, to discredit victims regardless of their age.

In contrast and in line with the hypotheses, as the child’s age increased, so did the probability that they would be asked a rape myth-consistent question (which drew upon myths related to force and resistance, motives to lie, precipitation, and character issues). These findings suggest some level of developmental sensitivity from defense attorneys when questioning children in court. Still, even questioning older children about rape myths is problematic because here questions likely overestimate the cognitive abilities of teenagers and older youth and may place unrealistic expectations on their ability to defend their own credibility. Myth-consistent questions are problematic for a child of any age, including teenagers, in that they draw upon misconceptions surrounding abuse and may unnecessarily challenge the child’s credibility for children who are *always* incapable of providing consent. Furthermore, rape myth lines of questioning attribute adult behaviors, like abuse precipitation, to children. These myths, even when used with adults, serve to justify abuse, defuse perpetrator blame, and are inconsistent with how many victims experience abuse. More problematic is their use, at an institutional level, with children. These myths never apply to children, who are incapable of precipitating their own abuse nor should they be expected to physically resist abuse. To illustrate, a defense attorney in our sample asked a 10-year-old girl the following question: “Okay. So he’s holding your arm. Were you, like, trying to get away?” Questions like these attribute blame to the child for not resisting abuse. Among rape myths, questions related to character seemed to be driving the main effect. That is, among questions that drew upon rape myths, those that reference a child’s character were more common among older children than younger children. That is, as the age of the child increased, so did the probability that a child would be asked a rape myth line of questioning addressing their character. Here we see attorneys tailoring the content of rape myth questions in ways that align with the child’s age and experiences. The use of rape myths in these cases is problematic, as even older children may be unable to identify and guard against attacks on their character. Questions that addressed the child’s character frequently referenced untrustworthy behaviors, promiscuity, and mental health issues. Again, it is likely these questions overestimate the developmental maturity of older children and teenagers and their ability to defend against attacks on their credibility. As rape myths influence case verdicts in important and meaningful ways (van der Bruggen & Grubb, 2014), it is likely that the use of these myths with children impacts the outcome of their cases.

Interestingly, there was no significant relationship between the child’s age and myths related to motives to lie, force and resistance, or precipitation. Here attorneys are indiscriminately asking children of every age about these rape myths. So, while young children may be statistically less likely to engage in promiscuous behavior like drinking with a defendant, attorneys are no less likely to ask about these precipitative behaviors. Young children in particular likely lack the developmental capacity to combat myths that draw upon precipitation for abuse, specifically. Furthermore, it is possible that the qualitative character of questions about specific CSA myths and specific rape myths may be related to age, even if the probability of these questions is not. Indeed, prior research suggests that the content of specific myth questions differs with the child’s age (Denne et al., 2023; St. George, Denne, et al., 2022).

### Limitations and Future Directions

The present work draws upon criminal trials from a single jurisdiction in the United States. As such, the results may not be widely generalizable to other jurisdictions or countries who use different procedures or legal structures to conduct CSA trials. Still, examinations of attorney questioning across numerous jurisdictions both inside and outside the United States yield fairly consistent and comparable patterns when examining both questioning style and, in some cases, questioning content in CSA cases (see Denne et al., 2020; Szojka et al., 2017). As such, these results add data that may provide a foundation for exploring developmental considerations in the content of attorney questioning beyond the current jurisdiction. Furthermore, we acquired our data through contacting and paying court reporters to share with us trial testimony they had transcribed. It is certainly possible that the cases they shared with us are substantially different in some way from the cases that we did not receive, introducing bias into our dataset. Notably, cases in our sample had a higher rate of conviction compared to all CSA cases for which we sought testimony. Defense attorneys’ use of CSA and rape myth-related questioning may have been even more prevalent in cases resulting in hung juries or acquittals. Unfortunately, testimonies from these cases were less likely to be transcribed and shared.

The focus of the present project was to examine age differences in defense attorney strategies when employing myths about sexual violence. We did not examine how children respond to these attacks on their credibility as that work lies beyond the scope of the present article. Still, valuable insight could be gained through examining how children interpret questions that capitalize on myths related to their disclosure, behavior, and character. In prior work, researchers suggest that children’s resistance to subtle questions with false implied meaning increases with age (Wylie et al., 2022). We might expect the same trajectory here, where children’s ability to resist and combat subtle myth-consistent questions may increase with age. In the future, researchers could examine whether children can identify these questions, and more importantly, how they respond to them in court.

Furthermore, we did not examine prosecutorial strategies or how they combat the use of rape myths by the defense. As prosecutors and defense attorneys have different motivations, we did not expect prosecutors to employ rape myths when questioning their witness. Still, it will be important to examine, through future work, how prosecutors respond to rape myths raised by the defense when questioning child victims. Prior work finds that with adult rape victims, prosecutors rarely addressed or challenged the defense’s use of rape myths (Temkin et al., 2018). This serves as a missed opportunity to educate the jury on misconceptions and false assumptions about sexual violence (Temkin et al., 2018). The same may be true in cases of sexual violence against children.

Through the present line of work, it was also possible that how old a child appeared may be more important than how old they actually were in predicting myth-consistent questions in defense attorney strategies. This may be particularly true for teenagers who are viewed as more adult-like. Researchers in the field of juvenile interrogations suggest that police officers view teenagers as adult-like and fail to apply knowledge of child development to how they question teenagers (Meyer, & Reppucci, 2007). Such may be true of defense attorneys as well, whose motivation to incriminate the witness may be similar to police officers’ motivation to incriminate a suspect. In this way, how old a child appears may predict the defense attorneys’ strategies and jurors’ perceptions of credibility more strongly than their biological age. Indeed, researchers suggest that jurors are not aware of the level of developmental sensitivity of questions asked to children in court, an effect that may be exacerbated for teenagers who appear physically older than they are developmentally (Cashmore & Trimboli, 2006).

Finally, our work is limited in scope. We did not examine differences in how attorneys question children based on the race, ethnicity, or gender of the child. It has been well documented that race plays a role in the investigation and prosecution of child maltreatment (Lane & Seltzer, 2023). It is possible then that a child’s race impacts how attorneys talk to them in court and the kinds of questions they use to credit or discredit the child’s report. Furthermore, our sample is primarily female as CSA disproportionately impacts females (Finkelhor, 1994). It is possible that the attorneys employ rape myths differently with male and female victims. Our sample of male victims was too small to systematically explore these potential differences, though we did control for these differences in our analyses. The interplay of race, gender, and age in how attorneys employ rape myths is an important avenue for future research but rests beyond the scope of the current article.

## Conclusion

Age-related differences in children’s abilities to interpret questions and communicate their experiences make both the content and structure of attorney questioning incredibly important for facilitating detailed, accurate, and reliable reports. While older children have more advanced cognitive and linguistic abilities, defense attorney questions may overestimate their abilities to interpret, understand, and defend against questions which draw upon myths related to sexual violence. The present project serves to expand upon St. George, Denne, et al. (2022) and Denne et al. (2023) to examine quantitative differences in the frequency with which defense attorneys employ rape and CSA myths in CSA cases across children of different ages. We found that attorneys used these myths strategically and in ways that were, at times, age sensitive and plausible. The probability that a child would receive a rape myth-consistent question increased with the child’s age, reflecting a sensitivity to young children’s limited development. Still, these myths are problematic, even when used with older children, as they highlight children’s presumed autonomy and precipitation. Older children similarly may be unable to defend their own credibility, though perhaps to a lesser extent.

To facilitate rich and accurate reports of maltreatment from children, attorneys’ questioning structure and content must be developmentally appropriate. Our research suggests that defense attorneys may be employing developmentally sensitive strategies when questioning young children, through using fewer rape myth-consistent questions with this age group. However, our findings also suggest a failure to apply developmentally sensitive questioning practices when questioning teenagers in court through the more frequent use of questions that draw upon myths related to sexual violence among adults. The use of rape myths with teenagers capitalizes on perceptions of them as adult-like and neglects to acknowledge their developing autonomy, agency, and legal ability to give consent. Researchers have established that when teenagers are questioned like adults, their responses are more likely to contain inconsistencies and miscommunications, undermining their credibility in court (Andrews & Lamb, 2017). Indeed, children’s responses are more informative when they are questioned in ways that are developmentally appropriate (Cashmore, 1992). To optimize children’s performance in court, both style and *content* of attorney questions should reflect developmental ability, even for older children, to facilitate clear communication and accurate reports. Our research suggests that the use of rape myths in court inappropriately attributes adult-like behavior, motives, agency, and autonomy to children.

Our study’s findings elucidate a disconnect between developmental research and defense attorney question content. Based on these findings, we suggest prosecutors be particularly sensitive to the use of myths with older children and teenagers, with whom defense attorneys are more likely to employ rape myths. In the future, researchers should address how prosecutors can effectively respond to attacks on the child’s credibility through objections or counterquestions in redirect examination.
